# Reliability and validity of computer vision‐based markerless human pose estimation for measuring hip and knee range of motion

**DOI:** 10.1049/htl2.70002

**Published:** 2025-01-31

**Authors:** Thomas Hellstén, Jari Arokoski, Jonny Karlsson, Leena Ristolainen, Jyrki Kettunen

**Affiliations:** ^1^ Faculty of Medicine University of Helsinki Helsinki Finland; ^2^ School of Engineering Culture and Wellbeing Arcada University of Applied Sciences Helsinki Finland; ^3^ Department of Internal Medicine and Rehabilitation Division of Rehabilitation Helsinki University Hospital Helsinki Finland; ^4^ Orton Orthopaedic Hospital Helsinki Finland; ^5^ Graduate School and Research Arcada University of Applied Sciences Helsinki Finland

**Keywords:** computer vision, patient rehabilitation, reliability, telemedicine

## Abstract

Telerehabilitation requires accurate joint range of motion (ROM) measurement methods. The aim of this study was to evaluate the reliability and validity of a computer vision (CV)‐based markerless human pose estimation (HPE) application measuring active hip and knee ROMs. For this study, the joint ROM of 30 healthy young adults (10 females, 20 males) aged 20–33 years (mean: 22.9 years) was measured, and test–retests were assessed for reliability. For validity evaluation, the CV‐based markerless HPE application used in this study was compared with an identical reference picture frame. The intraclass correlation coefficient (ICC) for the CV‐based markerless HPE application was 0.93 for active hip inner rotation, 0.83 for outer rotation, 0.82 for flexion, 0.82 for extension, and 0.74 for knee flexion. Correlations (*r*) of the two measurement methods were 0.99 for hip‐active inner rotation, 0.98 for outer rotation, 0.87 for flexion, 0.85 for extension, and 0.90 for knee flexion. This study highlights the potential of a CV‐based markerless HPE application as a reliable and valid tool for measuring hip and knee joint ROM. It could offer an accessible solution for telerehabilitation, enabling ROM monitoring.

## Graphical abstract

The aim of this study was to evaluate the reliability and validity of a computer vision (CV)‐based markerless human pose estimation (HPE) application measuring active hip and knee range of motions (ROM). This study highlights the potential of a CV‐based markerless HPE application as a reliable and valid tool for measuring hip and knee joint ROM

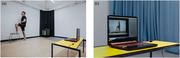

## 1 INTRODUCTION

For professionals in the healthcare sector, measuring joint range of motion (ROM) is vital in daily practice for the assessment and treatment of joint disorders [1]. Such measurement provides objective information about factors influencing the functioning of the joints and helps in assessing a patient's ability to perform daily activities, such as walking, running, or lifting [2], establish goals, and follow up on the progress of their treatment [3]. Therefore, information about joint ROM must be accurate [4]. Restriction or excessive joint ROM can be indicative of various disorders, such as osteoarthritis [5], joint deformities [6], or ligament injuries [7].

Different methods can be used to measure joint ROM. Universal goniometry is the most commonly used method by healthcare professionals [8] and it has been reported to be very close to the golden standard (radiographic image) in measuring ROM in the knee joint [9, 10]. A goniometer is accessible, low‐cost, portable, and user‐friendly; however, it requires the use of both hands by professionals, making it challenging to stabilize other body segments while measuring ROM [11]. Other methods commonly used by healthcare professionals to assess ROM are different inclinometers [11, 12], digital goniometers [4] and visual estimation [13]. Inclinometers are reliable for measuring certain joint angles, demonstrating good inter‐rater reliability; however, accurate and consistent positioning at the zero point is crucial to avoid measurement errors [14]. Visual estimation is quick, easy to use, and shows strong agreement with universal goniometer measurements when performed by an experienced healthcare professional [13]. The choice of method for measuring ROM depends on the joint being assessed and the availability of equipment [4], highlighting the need for novel approaches to overcome existing limitations.

The use of telerehabilitation (TR) by healthcare organizations has increased in the last few years [15, 16, 17]. TR promotes equality by offering easier access to healthcare to people who live, for example, in rural areas, by reducing travel and waiting times [18, 19]. TR can be a more cost‐effective method than clinical methods, and it provides opportunities for regular joint and posture assessments [20], offering important information and feedback about patients’ progress in treatment [21, 22]. According to some evidence, assessment that requires hands‐on interaction, such as specific measures such as ROMs is demanding to perform accurately with TR methods [23]. However, it has been discussed that developing TR assessment methods is important and seems to be feasible [20, 24].

A potential and novel method of implementing assessment in TR is computer vision (CV)‐based markerless human pose estimation (HPE) [20, 25], as the only technical equipment a patient needs in this method is a computing device (computer, tablet, smartphone) with an integrated camera. For tracking and analysing human motion, CV has been a significant and highly regarded research topic for several years [26]. To achieve accurate joint ROM measurements with CV‐based markerless HPE, joints must be accurately localized. Dense human pose estimation (DensePose) has become a promising technique for CV‐based markerless HPE joint ROM measurement due to its ease of use and accuracy. DensePose focuses on human anatomy, and the technique is designed to map two‐dimensional (2D) human images onto the three‐dimensional (3D) surface of the human body [27]. However, DensePose technique remains challenging on different background, hidden views, scale variations, and postural diversity [28]. Usually, CV‐based HPE motion analyses use marker‐based approaches that require the installation of reflective material on certain key points of the body, such as hip, knee, or shoulder joints. This limitation makes routine use of motion analysis systems impractical, as they require significant technical preparations prior to analysing motion. A 3D CV system such as Vicon has been used as the golden standard in the field of CV [29]; however, precisely calibrated equipment with several cameras requires high costs. Human motion analysis could also benefit from novel approaches, such as metaheuristic optimization methods [30], which can enhance the modelling and interpretation of complex motion patterns. Additionally, more traditional movement intensity data from accelerometers [31] can provide valuable insights, complementing advanced computational methods by offering direct measurements of motion dynamics. Nevertheless, they often require additional wearable equipment, which can make their use less practical compared to CV‐based markerless HPE, where users can leverage devices they already own.

In continuation of the previous article of the researchers of this study about the potential use of CV‐based markerless HPE in rehabilitation [24], this study took place. The aim was to evaluate the reliability and validity of a CV‐based markerless HPE (DensePose) application for measuring active hip flexion, extension, inner and outer rotation, and knee flexion. In this study, reliability was defined as the consistency of measurements across repeated CV‐based markerless HPE tests, while validity referred to the agreement between measurements obtained by the CV‐based markerless HPE application and a reference picture. The hypothesis for this study was that a CV‐based markerless HPE is a reliable and valid method for measuring active hip and knee joint ROM.

## 2 MATERIALS AND METHODS

### 2.1 Study design

**FIGURE 1:**
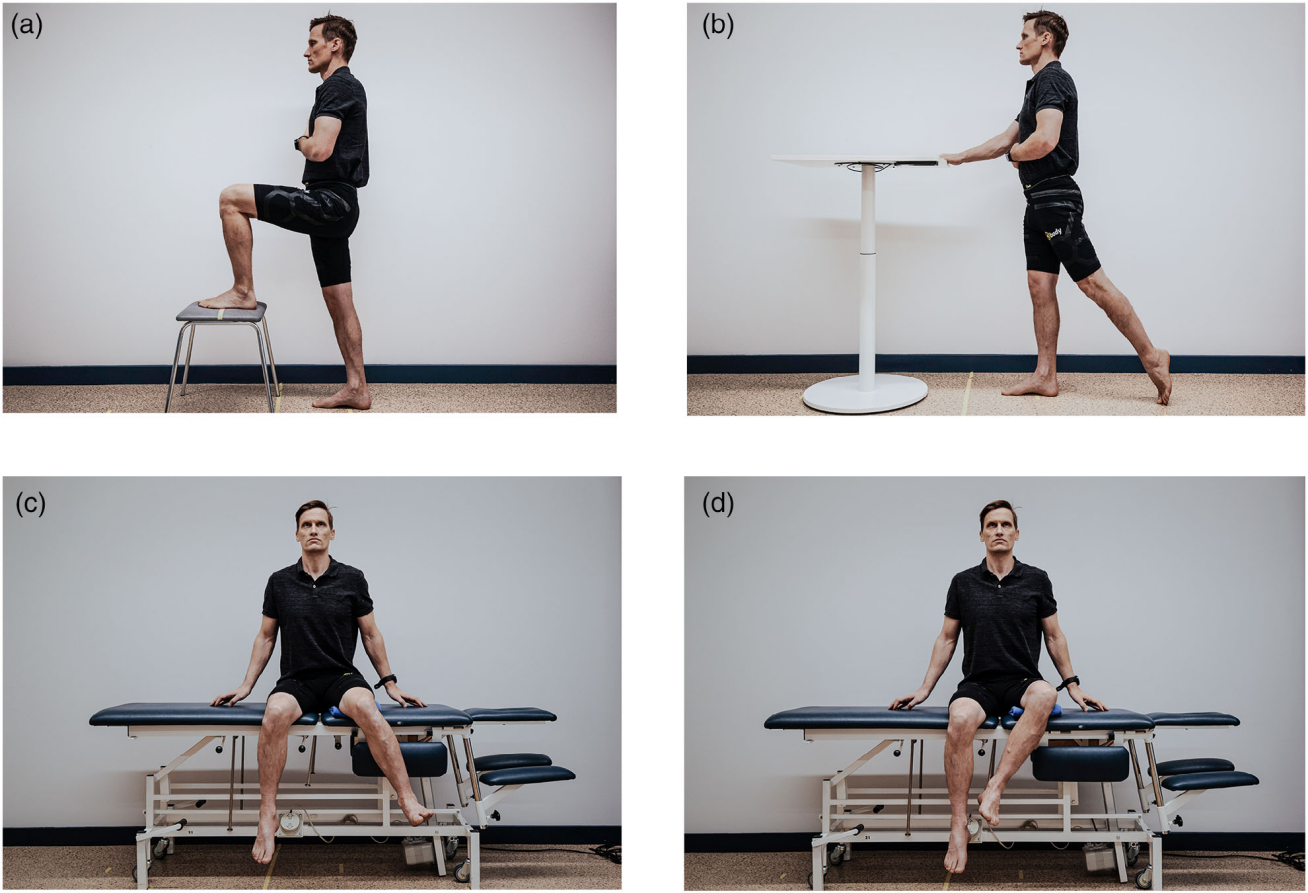
Measurement protocol: (a) Active flexion of hip and knee joint; (b) active extension of hip joint; (c) active inner‐ and (d) outer rotation of hip joint.

**FIGURE 2:**
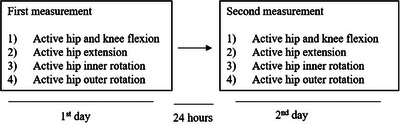
Study design, one participant was measured two times with an interval of 24 h. Joint angles were registered from the CV‐based markerless HPE application and an identical reference picture frame.

This is a methodological study to evaluate the reliability and validity of a CV‐based markerless HPE application built on DensePose for measuring active hip flexion, extension, inner and outer rotation, and knee flexion (Figure 1.). Written consent was obtained from all the voluntary participants before conducting the study, and they were informed that the current protocol had been approved by the research ethics committee of the Faculty of Medicine at the University of Helsinki (no. 1/2023). The measurements were performed twice, with an interval of 24 h, to evaluate the reliability of the CV‐based markerless HPE application. Validity was evaluated by comparing joint angles between the CV‐based markerless HPE application (automatically measured joint angle) and an identical reference picture frame (manually measured joint angle) (Figure 2). The study was conducted and evaluated according to the COnsensus‐based Standards for the selection of health Measurement INstruments (COSMIN) [32] pathway and reported according to principals of Strengthening the Reporting of Observational Studies (STROBE) Statement [33].

### 2.2 Participants

An information letter was mailed to students and personnel from Arcada University of Applied Sciences, Helsinki, in April 2023. Inclusion criteria were: healthy voluntary participants, who were 18 years of age or older, understood Swedish to follow the instructions, and were able to walk without any aid. Exclusion criteria were as follows: individuals having symptoms or pain in the lower limbs or lower back and who had required a visit to a physician in the last month before the study or diagnosis of neurological disease. Finally, 30 voluntary participants were recruited. The study participants (*N *= 30) were healthy young adults (10 females, 20 males) aged between 20 and 33 years (mean: 22.9 years), as shown in Table 1.

**TABLE 1:** Characteristics of the study participants.

Participants (*n*)	Age, years; mean (SD)	Length (cm); mean (SD)	Weight (kg); mean (SD)	BMI; mean (SD)
Total (30)	22.9 (2.8)	177.5 (10.6)	77.3 (14.1)	24.4 (2.9)
Female (10)	22.9 (3.8)	165.8 (3.7)	66.2 (9.2)	24.1 (4.0)
Male (20)	22.9 (2.2)	183.3 (7.6)	82.9 (12.8)	25.6 (2.7)

*Note*: *n*, number of participants; SD: standard deviation; cm, centimeter; kg, kilogram; BMI, body mass index (kg/m^2^).

### 2.3 CV‐based markerless HPE

Based on our literature review and technical testing, a CV‐based markerless HPE application using a single camera was developed for this study based on a human pose estimation system called DensePose [24, 27]. DensePose uses a region‐based convolutional neural network (R‐CNN) to map all pixels of a red, green, and blue (RGB) colour model image associated with a human to a 3D surface of the human body. Based on these 2D to 3D mappings, known as dense correspondences, the posture of the person is estimated. DensePose is trained on a large‐scale ground‐truth dataset called DensePoseCOCO [27], with manually annotated correspondences of 2D images to 3D surfaces on 50,000 images. DensePose takes one image as an input and, in addition to the dense correspondences, produces an output image marked with the 2D coordinates of key points of the human body, including the ankle, knee, hip, and shoulder joints.

**FIGURE 3:**
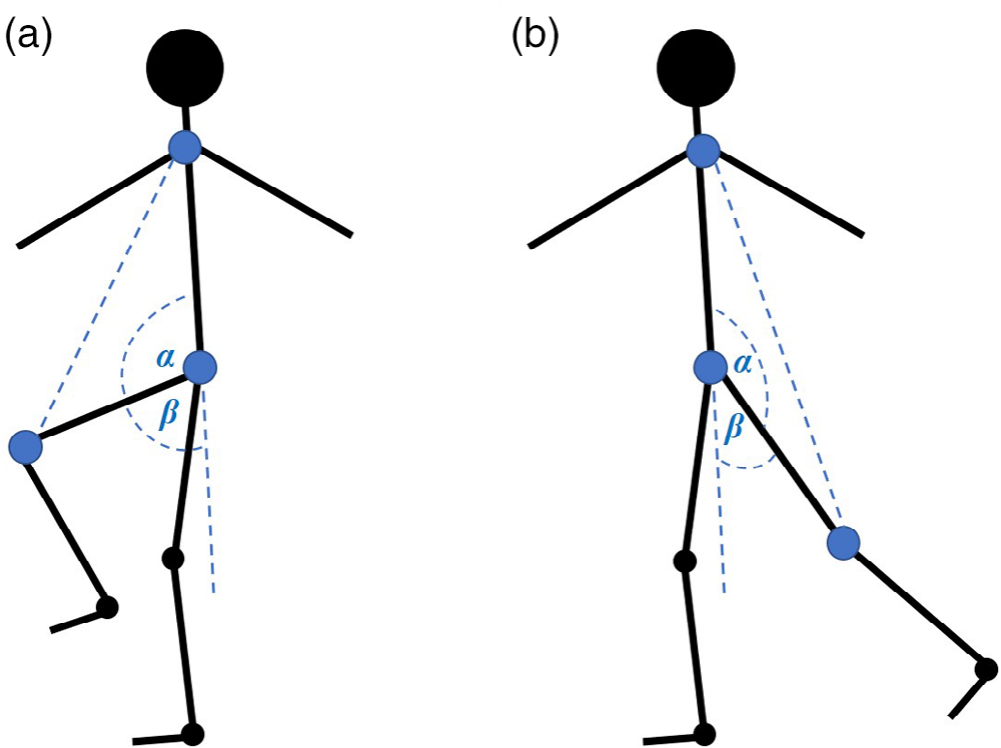
Key points and angles of interest for measuring hip (a) flexion and (b) extension. The angle is calculated as *β = *180* − α*. Blue dots show key points from a lateral view, the middle of the knee joint, hip joint, and shoulder joint.

To measure hip flexion and extension, the pixel coordinates of the knee, hip, and shoulder joints were first used to form a triangle. The angle *α* between the hip‐knee line and the hip‐shoulder line was then calculated by applying the law of cosines. Finally, the hip flexion and extension angle were calculated as *β* = 180 − *α*. The key points and angles of interest for measuring hip flexion and extension are shown in Figure 3.

**FIGURE 4:**
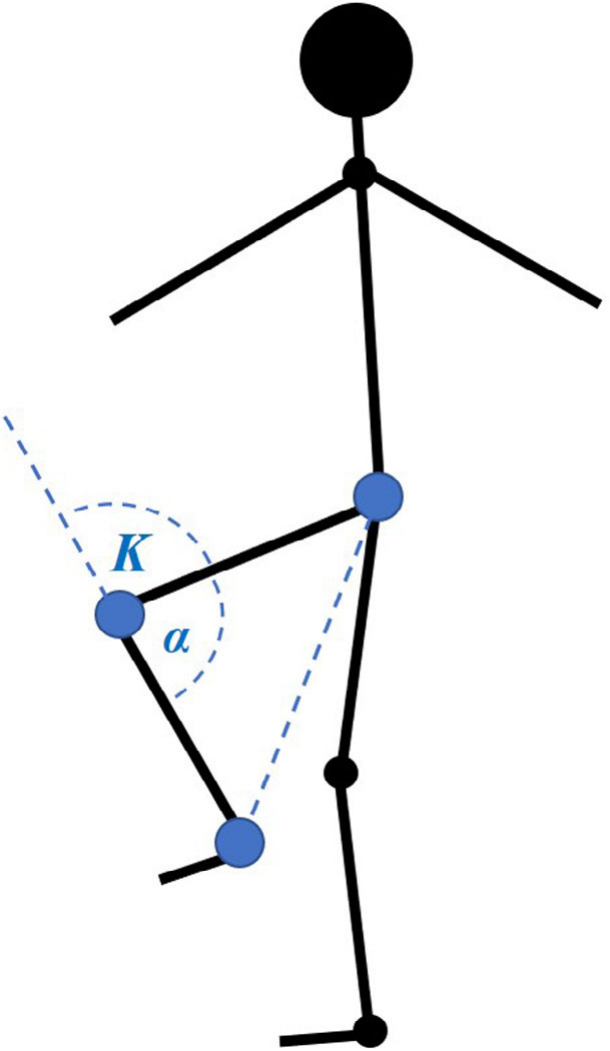
Key points and angles of interest for measuring knee range of motion. The angle is calculated as *β = *180* − α*. Blue dots show key points from a lateral view: the middle of the ankle joint, knee joint, and hip joint.

The knee angle was measured in a similar way to the hip, although in this case, the hip, knee, and ankle key points were used to formulate a triangle, and *α* was the angle between the hip–knee and knee–ankle lines (Figure 4).

**FIGURE 5:**
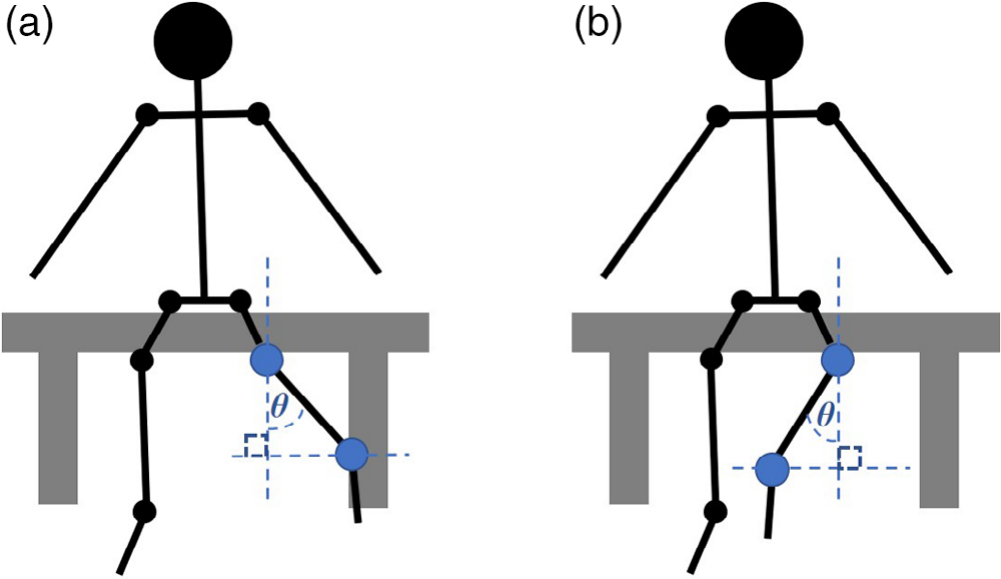
Key points and angles of interest for measuring hip (a) inner and (b) outer rotation. Angle *θ* is calculated using trigonometrical functions. Blue dots show key points from the frontal view, the middle of the ankle joint, and the knee joint.

To measure the inner and outer hip rotation angle *θ*, it was ensured that the camera was in a horizontal position. The application then captured the pixel coordinates of the knee and ankle joints for each frame. From the knee and ankle joints and the vertical pixel line from the knee joint downward, a right‐angle triangle was formed. Finally, the rotation angle *θ* was calculated using trigonometrical functions. The key points and angle of interest observed while measuring hip inner and outer rotation are shown in Figure 5.

**FIGURE 6:**
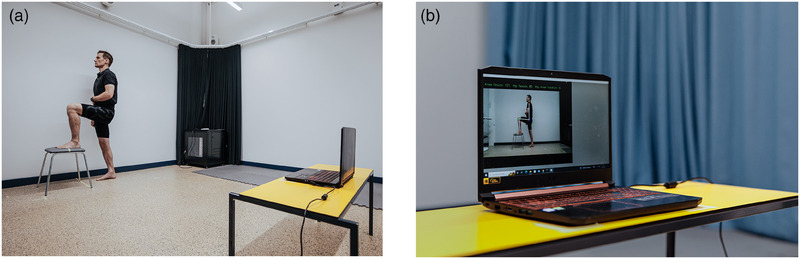
Photograph of (a) test setup when a participant performs active hip flexion and (b) computer screen view of our CV‐based markerless HPE application.

When running the CV‐based markerless HPE application, the joints and the hip flexion, extension, rotation, and knee flexion angles were shown frame by frame on the screen in real‐time. A save button was included, allowing the user to save the measured hip and knee angle to a log file at any given time, along with the video output frame on which the CV‐based markerless HPE application measured the angles. These output frames were used as reference pictures when analysing the validity of the hip and knee ROM measurements. During the tests, the positions of the computer, web camera, and participants were standardized. The measurement values were expressed in whole degrees. The computer used was Acer Nitro 5 with an integrated webcam (720p resolution and 30 frames per second sampling). The test setup is shown in Figure 6.

### 2.4 Reference picture

**FIGURE 7:**
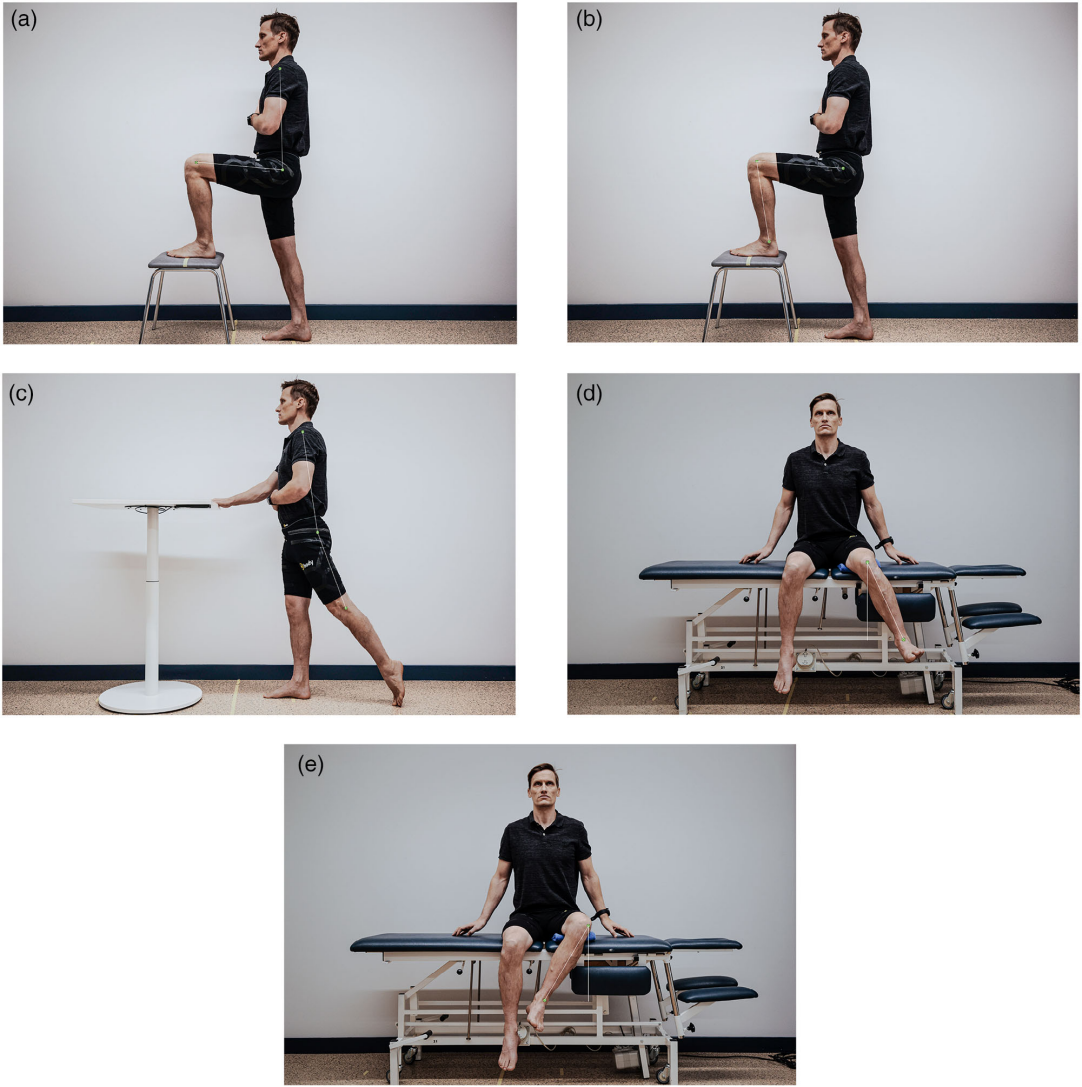
Reference picture with manually drawn markers for (a) active flexion of hip, (b) active flexion of knee joint, (c) active extension of hip joint, (d) active inner‐ and (e) outer rotation of hip joint.

Each reference picture generated as a result of pressing the Save button in the CV‐based markerless HPE application was a direct output from the web camera. The key points detected by the CV‐based markerless HPE application were not visible in the reference picture. To calculate active hip flexion and extension, markers were manually drawn on the reference picture as accurately as possible by an experienced physiotherapist on the left greater trochanter of the femur, lateral epicondyle of the femur, and middle of the humeral head. To calculate active inner and outer rotation in the hip joint, markers were drawn on the reference picture at the midpoint of the patella and the centre of the talocrural articular space. To calculate active flexion in the knee joint, markers were drawn on the greater trochanter of the femur, lateral epicondyle of the femur, and lateral malleolus of the fibula. Lines were drawn between markers to calculate the joint angles, as seen in Figure 7. Marker points were based on bony landmarks used when using a universal goniometer in physiotherapy [34]. The measurement values were given in degrees, with intervals of 1°.

### 2.5 Procedure

Before testing, the participants’ demographic data, weight, and length were measured, and body mass index (BMI = weight in kilograms divided by height in meters squared, kg/m^2^) was calculated. Environmental influences were standardized by taking the measurements in the same room, with the same light, temperature, position of computer, and web camera at a height of 55 cm and distance of 2.05 m from the participant (Figure 6). Reliability was measured through a test–retest procedure by the same test leader (24 h between the tests), and validity was tested with measurements during the first measurement with the CV‐based markerless HPE application and the reference picture (video output frame). The measurements were conducted on all 30 participants by two well‐trained, nearly graduated physiotherapist students on the left hip and knee joint in a standardized order: active flexion in the hip and knee joint, active extension in the hip joint, and active inner and outer rotation in the hip joint. No warm‐up period was included before testing. To become familiar with the test procedure, the instructions were read from a test manual and showed by the test leader (physiotherapist student). Test manual can be seen in Appendix. All measurements were recorded in a blinded manner, and the CV‐based markerless HPE application joint angle was saved to a log file with a time stamp and kept secret until the reference picture angle was calculated. All results were then saved in one file.

### 2.6 Statistical analysis

Descriptive statistics for each group were analysed and reported in the results, with their mean and standard deviation (SD). Test–retest reliability was evaluated with the intraclass correlation coefficient (ICC) with a two‐way random effect model and 95% confidence interval (CI), using the first and second CV‐based markerless HPE application measurements. ICC values were classified by Landis & Koch (1997) as follows: 1.00–0.81 as almost perfect, 0.80–0.61 as substantial, 0.60–0.41 as moderate, 0.40–0.21 as fair, and 0.20–0 as slight [35]. The standard error of measurement (SEM) was calculated using the formula $SEM=S\times \sqrt{1-ICC}$ for absolute reliability, where *S* = the SD of first and second CV‐based markerless HPE application measurement scores. Thereafter, the minimal detectable change (MDC) was calculated using the formula MDC=SEM×√2×1.96 [36].

Validity was established with Pearson's correlation analysis to compute the correlation between the CV‐based markerless HPE application and the reference picture (first measurement), and the Bland–Altman plot analysis was used to estimate the agreement between the two methods [37]. For correlation analysis, the following classification was used: 1.00–0.90 as very strong, 0.89–0.70 as strong, 0.69–0.50 as moderate, 0.49–0.30 as weak, and 0.29–0 as very weak [38]. Statistical analysis was performed using SPSS software (IBM Corp. Released 2022; IBM SPSS Statistics for Windows, Version 29.0. Armonk, NY: IBM Corp.) and Microsoft Excel Ver. 19.

## 3 RESULTS

The results showed almost perfect reliability for the CV‐based markerless HPE application in all movement directions in the hip joint (active flexion, active extension, active inner rotation, and active outer rotation), and the ICC values ranged from 0.93 to 0.82 in the hip joint. The highest ICC values were seen in active hip inner rotation (ICC = 0.93) and the lowest in active knee flexion (ICC = 0.74). Slightly low values were seen for SEM, and the values ranged from 0.6 (for active hip inner rotation) to 1.0 (for active knee flexion). The lowest MDC value was 1.6 for active hip inner rotation, and the highest was 3.2 for active hip outer rotation. More detailed results can be seen in Table 2.

**TABLE 2:** The reliability of CV‐based markerless HPE application in hip and knee joint.

Movement direction	ICC (95%CI)	SEM	MDC
Hip			
Active flexion	0.82 (0.63–0.92)	0.8	2.1
Active extension	0.82 (0.62–0.91)	0.8	2.1
Active inner rotation	0.93 (0.86–0.97)	0.6	1.6
Active outer rotation	0.83 (0.61–0.92)	0.8	3.2
Knee			
Active flexion	0.74 (0.45–0.87)	1.0	2.7

*Note*: CI: confidence interval; CV: computer vision, HPE: human pose estimation, ICC: intraclass correlation coefficient, MDC: minimal detectable change, SEM: standard error of measurement.

**FIGURE 8:**
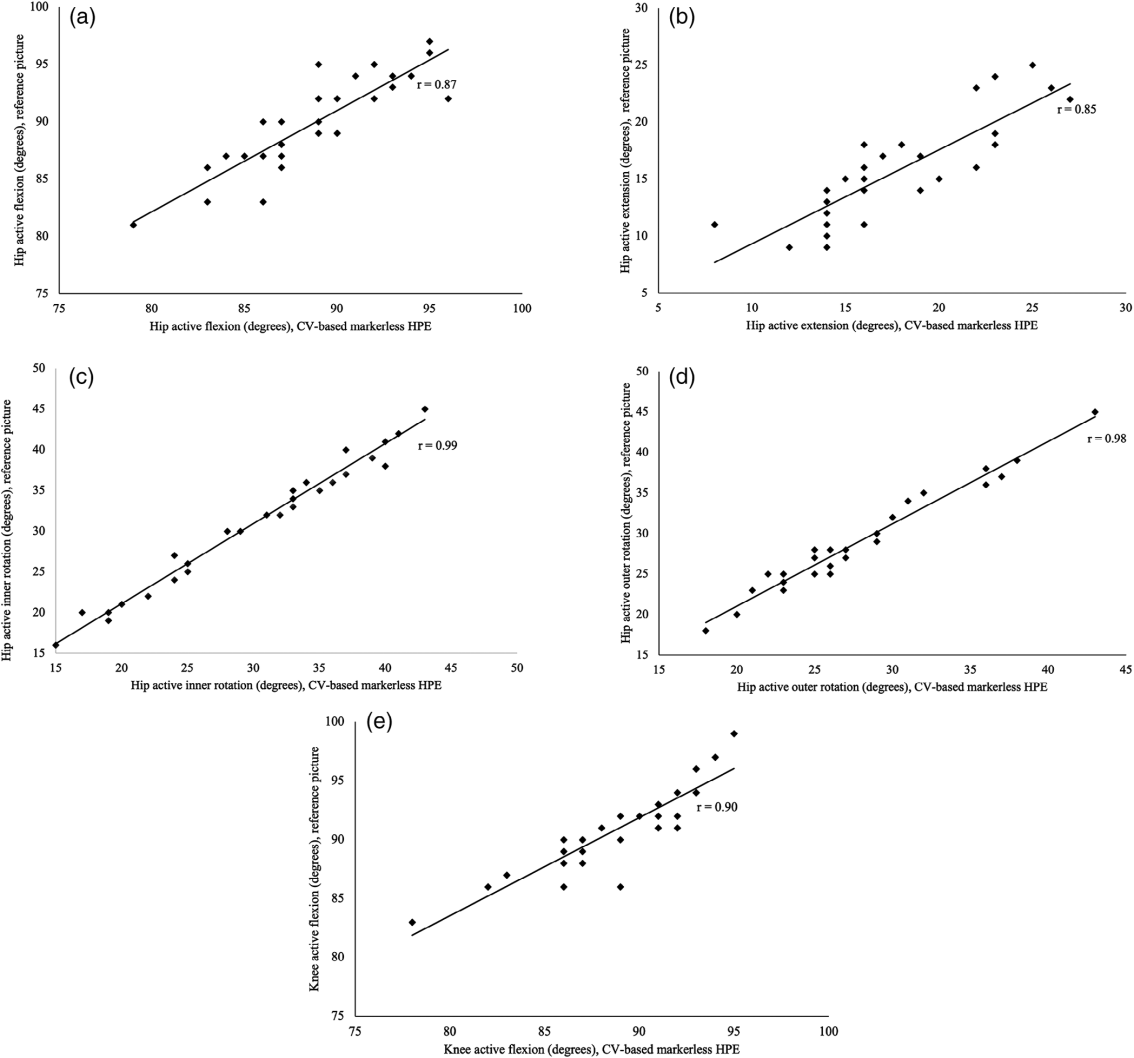
Correlation between our CV‐based markerless HPE application and the reference picture for (a) hip active flexion, (b) hip active extension, (c) hip active inner rotation, (d) hip active outer rotation and (e) knee active flexion, *r* = Person's correlation coefficient.

**FIGURE 9:**
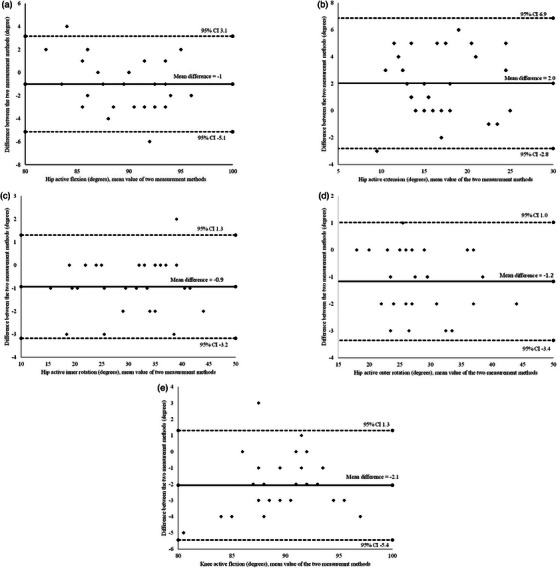
Bland–Altman plots for (a) hip active flexion, (b) hip active extension, (c) hip active inner rotation, (d) hip active outer rotation and (e) knee active flexion. The outer lines represent 95% limits of agreement. The middle line represents the mean of the differences between the two measurement methods.

The correlation (Person's *r* value) between the CV‐based markerless HPE application and the reference picture ranged from 0.99 to 0.85 and was very strong in three out of five directions. The correlation was highest in active hip inner rotation and lowest in active hip extension (Figure 8). The mean difference (degrees) between the two methods was lowest in active hip inner rotation (−0.9°), with one outlier outside the limits of agreement. The highest mean difference (−2.1°) was in active knee flexion, with one outlier outside the limits of agreement. Detailed results are illustrated in Figure 9.

## 4 DISCUSSION

This study analysed the reliability and validity of a CV‐based markerless HPE application for the measurement of hip and knee joint ROM. The results showed nearly perfect reliability in active hip flexion, active hip extension, active hip inner and outer rotation, and substantial reliability in active knee flexion. Correlations between our CV‐based markerless HPE application and the reference picture were very strong in more than one‐half of the joint angles and strong in two out of five joint angles. However, in knee flexion, the CV‐based markerless HPE application values tended to be smaller than in the reference picture.

Even if the ICC values were high in active hip flexion, extension, and inner and outer rotation, and slightly lower in knee flexion, the MDC values were relatively high (>2°) in four out of five directions. No established values exist on what acceptable accuracy is; however, a 2° MDC is acceptable in clinical settings, as universal goniometry, mostly used in the healthcare sector by clinicians as physiotherapist or physician, has shown a measure error of 6° in lower extremities [39].

The results of this study indicated good validity in almost all ROM measurements. However, in active hip flexion and extension, the correlations between the two methods were slightly lower. A reference photographic measurement [40] was used in this study; however, to obtain an exact joint angle, a radiograph image needs to be taken, which has been stated as the golden standard when measuring knee joint angle [9, 10]. Clinicians, such as physiotherapists and physicians, measure regular patients’ ROM to follow up on the treatment, for example, after a knee arthroplasty [41]. However, the radiograph measure is not frequently acceptable because of exposure to radiation [42]. Lower accuracy in active hip flexion and extension can be a result of difficulty for the CV‐based markerless HPE application algorithm to localize the joints (key points) to estimate correct angles, as Wang et al [25] also discussed in their study.

To measure hip or knee joint ROM, measurement methods and tools vary; however, universal goniometry is traditionally used in everyday practice by professionals [8], although it is not as exact method as radiograph measure [10, 39]; therefore, this method was not used for this study as a reference for the CV‐based markerless HPE application. Furthermore, CV‐based HPE using markers with one or several cameras was not used in this study, which would have required a major financial investment and is impractical in daily practice in the healthcare sector. Hence, an easy and reliable method, photographic measurement [40], was used as a reference for the CV‐based markerless HPE application measured angles. The reference picture was exactly the same picture frame from which the CV‐based markerless HPE application analysed the joint angle. There was no possibility of palpating bony landmarks for markers in the reference picture; however, they were visually estimated by an experienced physiotherapist. Furthermore, the lack of extension in the knee joint was not measured, as the participants were healthy, young, and had no acute injury in the lower extremity. However, a lack of knee extension decreases quadriceps contraction [4] and the ability of normal knee function in, for example, patients with knee osteoarthritis [5]. Future studies should evaluate CV‐based markerless HPE in patients with symptoms in the lower extremities.

The use of TR increased significantly under the COVID‐19 pandemic in daily practice, even if TR might be demanding to be used when healthcare professionals (physiotherapists) need hands‐on action [16, 17] as evaluation of joint ROM. Currently, the best way for healthcare professionals (physiotherapists) to provide TR for patients at a distance is through consultation, guidance and counselling [23]. In daily practice, patient intervention needs to include documentation from the beginning of the intervention to the end [43] to evaluate the effects of the intervention. Documentation must be well written, as it influences patient care [44]; however, it is also used to evaluate the cost of care and obtain statistics [45]. However, to be able to evaluate and document, for example, stiffness in the hip or knee joint after an injury with TR methods, may be challenging. Thereby, CV‐based markerless HPE can potentially be used in the future to analyse patient joint ROM at a distance, as they neither need hardware other than a computing device with a camera nor healthcare professional's expertise. Moreover, the use of CV‐based markerless HPE includes an increased possibility for regular measurement. Thereby, it can increase patient motivation and add to the treatment process [2]. To promote the widespread adoption of CV‐based markerless HPE applications, efforts should prioritize user‐friendly software interfaces, compatibility with existing clinical workflows, and rigorous validation across diverse environments. Additionally, comprehensive training resources for healthcare professionals and effective patient education will be crucial for facilitating their integration into routine care before implementation in daily practice.

### 4.1 Strengths and limitations

A strength of this study is the number of participants (*N* = 30). Errors were minimized using an optimal environment by standardizing the measurements at a clinic. In addition, a bright room was used with an ideal lightning system, positioned computer, web camera, and participant test position. However, it is obvious, as Hannik et al [20] noted, that as a CV‐based markerless HPE uses a camera from a computing device, it is influenced by the environment and technical competence of the patient. Errors in measurement can arise from the instrument used, the professionals involved, or variability in the performance of the patient [46]. Furthermore, this study is based on multidisciplinary collaboration between healthcare (physician/physiotherapist) and engineering professionals, which has been discussed as important in these kinds of development processes [25].

This study also has some limitations. The same test order was used when the joint angles were measured, and it may have influenced the results. By changing the test order, this limitation could have been eliminated; however, it was not done for practical reasons.

The current sample of healthy young adults with optimum weight (BMI 18.5–24.9), may limit the generalizability of findings to older adults or clinical populations. Future research should validate these results in diverse demographic groups, including older adults and in individuals with different disease conditions, to ensure broader applicability. It was found that lightning especially influenced how well the CV‐based markerless HPE application could detect and localize the body reference points (markers) to calculate joint ROM. Furthermore, participants’ joint angles were measured in an upright position as a consequence of a lack of training data for the computer (DensePose‐COCO dataset), which represented patients in different positions than upright (standing). In the future, this kind of technical challenge should be considered before CV‐based markerless HPE can be implemented in daily practice in the healthcare sector.

One limitation of this study is the absence of radiographic images, which are considered the gold standard for ROM measurements. However, ethical concerns regarding radiation exposure and resource constraints precluded their use. Future studies could investigate non‐invasive imaging alternatives, such as ultrasound or advanced magnetic resonance imaging techniques, to further validate CV‐based markerless HPE applications.

## 5 CONCLUSION

This study's CV‐based markerless HPE application showed highly repeatable results in the hip joint, active flexion, active extension, active inner rotation, and active outer rotation. In active knee flexion, the results indicated substantial repeatability. MDC values were relatively high in all directions; however, they were acceptable for healthcare professionals when measuring joint ROM in daily practice. The correlations between the two measurement methods were very strong in three out of five directions: active hip inner rotation, active hip outer rotation, and active knee flexion; however, in knee flexion, the CV‐based markerless HPE application values tended to be smaller than those in the reference picture. CV‐based markerless HPE can potentially be used to analyse joint ROM in hip and knee joints. To promote CV‐based markerless HPE adoption, efforts should focus on user‐friendly interfaces, workflow compatibility, rigorous validation, and providing training for healthcare professionals and education for patients to support integration into routine care.

## AUTHOR CONTRIBUTIONS

Thomas Hellstén, Jari Arokoski, Jonny Karlsson, Leena Ristolainen, and Jyrki Kettunen contributed to the conception and design of the work. Jonny Karlsson contributed to the CV‐based markerless HPE application. Thomas Hellstén, collected data, analysed data, wrote the first version of the manuscript. All authors participated in interpreting the data and revising critically on important part of the article and approved the final version of the manuscript.

## CONFLICT OF INTEREST STATEMENT

The authors declare no conflicts of interest.

## Data Availability

Data not available—participant consent.

## TEST MANUAL

### Active hip and knee flexion

Standing position instruction: ‘Bend your left hip and knee, and lift your midfoot on the line on the chair. Keep your back and head straight. Look straight forward on the mark on the wall (Figure 1a). Maintain this position for 3 s. I will just demonstrate the movement to you. Do you have any questions?’ After the participant had taken the position, the instructor counted three–two–one and saved the CV‐based markerless HPE application registered joint angle from the hip and knee flexion on the log file with a time stamp. The reference picture was saved on a computer with an identical time stamp from the same picture frame, as the joint angle was calculated using the CV‐based markerless HPE application. The saving procedure was the same for all measurements.

### Active hip extension

Standing position instruction: ‘Slightly touch the table with your right hand to maintain balance. Place the palm of your left hand on your stomach. Extend your left hip so long that your toes touch the floor. Do not bend your back, keep your head straight, and look forward on the mark on the wall (Figure 1b). Maintain this position for 3 s. I will just demonstrate the required movement to you. Do you have any questions?’

### Active hip inner and outer rotation

Sitting position instruction: ‘Rotate the left hip inward by bringing the left foot outward from the midline (Figure 1c), then rotate the hip outward by bringing the foot toward the midline (Figure 1d). Keep your pelvis in place as you perform the movement. Maintain both positions for 3 s. I will just demonstrate the required movement to you. Do you have any questions?’

## References

[1] Lind, V. , Svensson, M. , Harringe, M.L. : Reliability and validity of a digital goniometer for measuring knee joint range of motion. Meas. Phys. Educ. Exercise Sci. 26(3), 191–198 (2022)

[2] Hynynen, P. , Häkkinen, H. , Hännikäinen, H. , Kangasperko, M. , Karihtala, T. , Keskinen, M. , et al.: The corecompetences of a physiotherapist. Suomen Fysioterapeutit. https://www.suomenfysioterapeutit.fi/wp‐content/uploads/2018/04/CoreCompetencies.pdf (2016). Accessed Dec 2023

[3] Russell, T.G. , Jull, G.A. , Wootton, R. : Can the Internet be used as a medium to evaluate knee angle? Manual Ther. 8(4), 242–246 (2003) 10.1016/s1356-689x(03)00016-x 14559047

[4] Hancock, G.E. , Hepworth, T. , Wembridge, K. : Accuracy and reliability of knee goniometry methods. J. Exp. Orthop. 5(1), 46 (2018) 30341552 10.1186/s40634-018-0161-5 PMC6195503

[5] Liikavainio, T. , Lyytinen, T. , Tyrväinen, E. , Sipilä, S. , Arokoski, J.P. : Physical function and properties of quadriceps femoris muscle in men with knee osteoarthritis. Arch. Phys. Med. Rehabil. 89(11), 2185–2194 (2008) 18996249 10.1016/j.apmr.2008.04.012

[6] Sankar, W.N. , Laird, C.T. , Baldwin, K.D. : Hip range of motion in children: What is the norm? J. Pediatr. Orthop. 32(4), 399–405 (2012) 22584842 10.1097/BPO.0b013e3182519683

[7] Mendonça, L.D. , Ocarino, J.M. , Bittencourt, N.F.N. , Macedo, L.G. , Fonseca, S.T. : Association of hip and foot factors with patellar tendinopathy (Jumper's Knee) in athletes. J. Orthop. Sports Phys. Ther. 48(9), 676–684 (2018) 29792104 10.2519/jospt.2018.7426

[8] Cibere, J. , Thorne, A. , Bellamy, N. , Greidanus, N. , Chalmers, A. , Mahomed, N. , et al.: Reliability of the hip examination in osteoarthritis: Effect of standardization. Arthritis Rheum 59(3), 373–381 (2008) 18311750 10.1002/art.23310

[9] Gogia, P.P. , Braatz, J.H. , Rose, S.J. , Norton, B.J. : Reliability and validity of goniometric measurements at the knee. Phys. Ther. 67(2), 192–195 (1987) 3809242 10.1093/ptj/67.2.192

[10] Brosseau, L. , Tousignant, M. , Budd, J. , Chartier, N. , Duciaume, L. , Plamondon, S. , et al.: Intratester and intertester reliability and criterion validity of the parallelogram and universal goniometers for active knee flexion in healthy subjects. Physiother. Res. Int. 2(3), 150–166 (1997) 9421820 10.1002/pri.97

[11] Roach, S. , San Juan, J.G. , Suprak, D.N. , Lyda, M. : Concurrent validity of digital inclinometer and universal goniometer in assessing passive hip mobility in healthy subjects. Int. J. Sports Phys. Ther. 8(5), 680–688 (2013) 24175147 PMC3811733

[12] Hunter, D.J. , Rivett, D.A. , McKeirnan, S. , Smith, L. , Snodgrass, S.J. : Relationship between shoulder impingement syndrome and thoracic posture. Phys. Ther. 100(4), 677–686 (2019) 10.1093/ptj/pzz182 31825488

[13] Holm, I. , Bolstad, B. , Lütken, T. , Ervik, A. , Røkkum, M. , Steen, H. : Reliability of goniometric measurements and visual estimates of hip ROM in patients with osteoarthrosis. Physiother. Res. Int. 5(4), 241–248 (2000) 11129666 10.1002/pri.204

[14] Kolber, M.J. , Vega, F. Jr. , Widmayer, K. , Cheng, M.S. : The reliability and minimal detectable change of shoulder mobility measurements using a digital inclinometer. Physiother. Theory Pract. 27(2), 176–184 (2011) 20690872 10.3109/09593985.2010.481011

[15] Giesbrecht, E. , Major, M.E. , Fricke, M. , Wener, P. , van Egmond, M. , Aarden, J.J. , et al.: Telerehabilitation delivery in Canada and the Netherlands: Results of a survey study. JMIR Rehabil. Assist. Technol. 10, e45448 (2023) 36806194 10.2196/45448 PMC9989917

[16] Hellstén, T. , Arokoski, J. , Sjögren, T. , Jäppinen, A.‐M. , Kettunen, J. : The current state of remote physiotherapy in Finland: Cross‐sectional web‐based questionnaire study. JMIR Rehabil. Assist. Technol. 9(2), e35569 (2022) 35609305 10.2196/35569 PMC9177171

[17] Rausch, A.‐K. , Baur, H. , Reicherzer, L. , Wirz, M. , Keller, F. , Opsommer, E. , et al.: Physiotherapists' use and perceptions of digital remote physiotherapy during COVID‐19 lockdown in Switzerland: An online cross‐sectional survey. Arch. Physiotherapy 11(1), 18 (2021) 10.1186/s40945-021-00112-3 PMC8261812 34233763

[18] Damhus, C.S. , Emme, C. , Hansen, H. : Barriers and enablers of COPD telerehabilitation—A frontline staff perspective. Int. J. Chron. Obstruct. Pulmon Dis. 13, 2473–2482 (2018) 30154650 10.2147/COPD.S167501 PMC6103610

[19] Turolla, A. , Rossettini, G. , Viceconti, A. , Palese, A. , Geri, T. : Musculoskeletal physical therapy during the COVID‐19 pandemic: Is telerehabilitation the answer? Phys. Ther. 100(8), 1260–1264 (2020) 32386218 10.1093/ptj/pzaa093 PMC7239136

[20] Hannink, E. , Mansoubi, M. , Cronin, N. , Wilkins, B. , Najafi, A.A. , Waller, B. , et al.: Validity and feasibility of remote measurement systems for functional movement and posture assessments in people with axial spondylarthritis. Healthcare Technol. Lett. 9(6), 110–118 (2022) 10.1049/htl2.12038 PMC9731560 36514477

[21] Emedoli, D. , Alemanno, F. , Houdayer, E. , Brugliera, L. , Iannaccone, S. , Tettamanti, A. : Mobile application tool for remote rehabilitation after discharge from coronavirus disease‐19 rehabilitation unit. Healthcare Technol. Lett. 9(4‐5), 70–76 (2022) 10.1049/htl2.12033 PMC9535743 36225346

[22] Ceprnja, D. , Clark, T. , Young, J. , Lee, R. , Flynn, K. , Maka, K. : Evaluating experiences, usability and patient satisfaction with telehealth for tertiary outpatient physiotherapy services during COVID‐19: A mixed‐methods study. Physiother. Theory Pract. 39(9), 1929–1937 (2023) 35387568 10.1080/09593985.2022.2059423

[23] Hellstén, T. , Arokoski, J. , Sjögren, T. , Jäppinen, A.‐M. , Kettunen, J. : Remote physiotherapy in Finland—suitability, usability and factors affecting its use. Eur. J. Physiother. 25(6), 378–387 (2023)

[24] Hellsten, T. , Karlsson, J. , Shamsuzzaman, M. , Pulkkis, G. : The potential of computer vision‐based marker‐less human motion analysis for rehabilitation. Rehabil. Process Outcome 10, 11795727211022330 (2021) 34987303 10.1177/11795727211022330 PMC8492027

[25] Wang, X.M. , Smith, D.T. , Zhu, Q. : A webcam‐based machine learning approach for three‐dimensional range of motion evaluation. PLoS One 18(10), e0293178 (2023) 37871043 10.1371/journal.pone.0293178 PMC10593217

[26] Debnath, B. , O'brien, M. , Yamaguchi, M. , Behera, A. : A review of computer vision‐based approaches for physical rehabilitation and assessment. Multimedia Syst. 28(1), 209–239 (2022)

[27] Guler, R. , Neverova, N. : DensePose: Dense human pose estimation in the wild. In: Proceedings of the 2018 IEEE/CVF Conference on Computer Vision and Pattern Recognition, pp. 7297–7306. IEEE, Piscataway, NJ (2018)

[28] Guo, Y. , Gao, L. , Song, J. , Wang, P. , Xie, W. , Shen, H.T. : Adaptive multi‐path aggregation for human densepose estimation in the wild. In: Proceedings of the 27th ACM International Conference on Multimedia, pp. 356–364. Association for Computing Machinery, New York, NY (2019)

[29] Aughey, R.J. , Ball, K. , Robertson, S.J. , Duthie, G.M. , Serpiello, F.R. , Evans, N. , et al.: Comparison of a computer vision system against three‐dimensional motion capture for tracking football movements in a stadium environment. Sports Eng. 25(1), 2 (2022)

[30] Xu, D. , Zhou, H. , Quan, W. , Jiang, X. , Liang, M. , Li, S. et al.: A new method proposed for realizing human gait pattern recognition: Inspirations for the application of sports and clinical gait analysis. Gait Posture 107, 293–305 (2024) 37926657 10.1016/j.gaitpost.2023.10.019

[31] Dorris, H. , Oh, J. , Jacobson, N. : Wearable movement data as a potential digital biomarker for chronic pain: An investigation using deep learning. Phys. Act. Health 8(1), 83–92 (2024)

[32] Mokkink, L.B. , Prinsen, C. , Patrick, D.L. , Alonso, J. , Bouter, L.M. , De Vet, H. , et al.: COSMIN study design checklist for patient‐reported outcome measurement instruments. Gut 70, 139–147 (2019)

[33] von Elm, E. , Altman, D.G. , Egger, M. , Pocock, S.J. , Gøtzsche, P.C. , Vandenbroucke, J.P. : The strengthening the reporting of observational studies in epidemiology (STROBE) statement: Guidelines for reporting observational studies. Int. J. Surg. 12(12), 1495–1499 (2014) 25046131 10.1016/j.ijsu.2014.07.013

[34] Reese, N.B. , Bandy, W.D. : Joint Range of Motion and Muscle Length Testing, Elsevier Health Sciences, Amsterdam (2016)

[35] Landis, J.R. , Koch, G.G. : The measurement of observer agreement for categorical data. Biometrics 33(1), 159–174 (1977) 843571

[36] Donoghue, D. , Stokes, E.K. : How much change is true change? The minimum detectable change of the Berg balance scale in elderly people. J. Rehabil. Med. 41(5), 343–346 (2009) 19363567 10.2340/16501977-0337

[37] Bland, J.M. , Altman, D.G. : Statistical methods for assessing agreement between two methods of clinical measurement. Lancet 1(8476), 307–310 (1986) 2868172

[38] Hinkle, D.E. , Jurs, S.G. , Wiersma, W. : Applied Statistics for the Behavioral Sciences, Houghton Mifflin, Boston (2003)

[39] Boone, D.C. , Azen, S.P. , Lin, C.‐M. , Spence, C. , Baron, C. , Lee, L. : Reliability of goniometric measurements. Phys. Ther. 58(11), 1355–1360 (1978) 704684 10.1093/ptj/58.11.1355

[40] Kouyoumdjian, P. , Coulomb, R. , Sanchez, T. , Asencio, G. : Clinical evaluation of hip joint rotation range of motion in adults. Orthop. Traumatol.: Surg. Res. 98(1), 17–23 (2012) 22227606 10.1016/j.otsr.2011.08.015

[41] Calatayud, J. , Casaña, J. , Ezzatvar, Y. , Jakobsen, M.D. , Sundstrup, E. , Andersen, L.L. : High‐intensity preoperative training improves physical and functional recovery in the early post‐operative periods after total knee arthroplasty: A randomized controlled trial. Knee Surg. Sports Traumatol. Arthrosc. 25(9), 2864–2872 (2017) 26768606 10.1007/s00167-016-3985-5

[42] Frane, N. , Megas, A. , Stapleton, E. , Ganz, M. , Bitterman, A.D. : Radiation exposure in orthopaedics. JBJS Rev. 8(1), e0060 (2020) 31899700 10.2106/JBJS.RVW.19.00060

[43] Rosenbloom, S.T. , Denny, J.C. , Xu, H. , Lorenzi, N. , Stead, W.W. , Johnson, K.B. : Data from clinical notes: A perspective on the tension between structure and flexible documentation. J. Am. Med. Inf. Assoc. 18(2), 181–186 (2011) 10.1136/jamia.2010.007237 PMC3116264 21233086

[44] Adane, K. , Gizachew, M. , Kendie, S. : The role of medical data in efficient patient care delivery: A review. Risk Manag. Healthcare Policy 12, 67–73 (2019) 10.2147/RMHP.S179259 PMC6486797 31114410

[45] Lorkowski, J. , Maciejowska‐Wilcock, I. , Pokorski, M. : Overload of medical documentation: A disincentive for healthcare professionals. Med. Res. Innovation 1324, 1–10 (2021) 10.1007/5584_2020_587 33034843

[46] Russek, L. : Factors affecting interpretation of reliability coefficients. J. Orthop. Sports Phys. Ther. 34(6), 341–349 (2004) 15233396 10.2519/jospt.2004.0797

